# Ultrastructural Analysis of Rehydrated Human Donor Corneas After Air-Drying and Dissection by Femtosecond Laser

**DOI:** 10.3389/fmed.2021.787937

**Published:** 2021-12-21

**Authors:** Emilio Pedrotti, Erika Bonacci, Adriano Fasolo, Arianna De Rossi, Davide Camposampiero, Gary L. A. Jones, Paolo Bernardi, Flavia Merigo, Diego Ponzin, Giorgio Marchini, Andrea Sbarbati

**Affiliations:** ^1^Ophthalmology Unit, Department of Neurosciences, Biomedicine and Movement Sciences, University of Verona, Verona, Italy; ^2^Research Unit, The Veneto Eye Bank Foundation, Venice, Italy; ^3^Anatomy and Histology Section, Department of Neurosciences, Biomedicine and Movement Sciences, University of Verona, Verona, Italy

**Keywords:** dehydrated cornea, electron microscopy, femtosecond laser, light microscopy, rehydrated cornea

## Abstract

**Purpose:** To evaluate the efficiency of femtosecond laser (FSL) incision of rehydrated human donor corneas after air-drying and its effects on corneal structure.

**Methods:** We compared the rehydrated and fresh-preserved corneas by microscopy following Victus-Tecnolas FSL treatment for straight-edge anterior lamellar keratoplasty (ALK). The corneas were dehydrated at room temperature under a laminar-flow hood.

**Results:** To obtain the horizontal cut in rehydrated corneas, we increased the FSL pulse energy to 1.2 μJ from 0.80 μJ applied for the fresh corneas and obtained a clear-cut separation of the lamellar lenticule cap from the corneal bed. Light microscopy showed regular arrangement of stromal collagen lamellae, with spaces in between the fibers in the corneal stroma in the fresh and the rehydrated corneas, but the uppermost epithelial layers in the rehydrated corneas were lost. Transmission electron microscopy (TEM) revealed no signs of thermal or mechanical damage to the corneal structure. The epithelial basal membrane and Bowman's layer maintained their integrity. The epithelial basal layer and cells were separated by large spaces due to junction alteration in the rehydrated corneas. There were gaps between the lamellar layers in the stroma, especially in the rehydrated corneas. Keratocytes displayed normal structure in the fresh corneas but were devoid of microorganules in the rehydrated corneas. Minor irregularities were observed in the vertical incision and the horizontal stroma appeared smooth on scanning electron microscopy.

**Conclusion:** The corneal stroma of rehydrated corneas maintained morphology and integrity, while corneal cellular components were generally altered. When corneas are intended for FSL-assisted ALK, effective stromal bed incision is best achieved at a laser power higher than that currently adopted for fresh corneas.

## Introduction

Hypothermia and organ culture are the two most important storage methods employed by the American and European eye banks for the preservation of human donor corneas for up to 2 and 4 weeks, respectively (1, 2).

Longer storage periods may become necessary when there is an unexpected rise in supply or a fall in demand of corneal tissue, as has occurred during the COVID-19 pandemic (3, 4), during annual recess periods, or in low-income countries where appropriate healthcare structures and eye-banking frameworks are sometimes lacking.

The conventional preservation times of whole corneas and stromal lenticules can be lengthened by cryopreservation/vitrification (5), dehydration without freezing (6), glycerolization (7–9), lyophilization (or *freeze-drying*) (10–12), and sterilization by gamma irradiation (13).

Such methods, however, can disrupt tissue and cell structures (14, 15). Non-viable whole corneas or anterior stromal lenticules can be employed to treat corneal stroma diseases not involving the endothelial layer or for reconstructive indications, thus allowing subsequent visual grafting. Whereas the application of corneas after cryopreservation can result in poor graft quality (5), lyophilized or silica gel-dehydrated corneal lenticules have been successfully employed in treating epikeratophakia, corneal scars, and keratoconus without negative clinical outcome after keratoplasty (6, 12, 16).

With the introduction of the femtosecond laser (FSL) for corneal trephination, lamellar techniques have become an easier and more predictable choice to place lamellar cuts at the desired plane (17). FSL-assisted anterior lamellar keratoplasty (ALK) allows for a precise and controlled incision, which is key to the successful pneumatic dissection and graft-host interface, with superior visual recovery and/or reduced complications (18).

In this study, we tested the feasibility of air-drying and FSL-assisted dissection of rehydrated corneas to obtain an anterior lamellar graft, and analyzed the quality of the stromal surface at the horizontal and the side cuts by light and electron microscopy.

## Materials and Methods

### Donor Corneas

Human donor corneas were obtained from *Fondazione Banca degli Occhi del Veneto* (The Veneto Eye Bank Foundation, FBOV, Venice, IT). Written consent was obtained from the next kin of donor for the tissues evaluated as being unsuitable for therapeutic application, to be used for research purposes (Protocol no. CRT/19 rev. 02, 24 May 2018). The study adhered to the tenets of the Declaration of Helsinki.

The study materials were corneas with a clear and uncompromised stroma and without apparent irregularities, nonetheless unsuitable for transplantation because of donor serology or poor endothelial cell density. Four corneas were dehydrated after preservation in sterile *Storage* solution (MEM-Earle with HEPES 25 mM, antibiotics, dextran T500) at 31°C. Two corneas, used as controls (CTRL), were maintained in hypothermic storage at 2–6°C in a sterile *Cold* solution (MEM-Earle with HEPES 25 mM, antibiotics, dextran T500); conditions that do not influence the proper physical characteristics of the corneas, though the cornea swells to about two times its normal thickness and the number of layers of epithelial cells are reduced (19).

### Dehydration and Rehydration Process

The corneas were dehydrated in the eye bank laboratory by positioning them on a Teflon base, endothelial side up, and then air-dried overnight (12–15 h) at room temperature under a laminar-flow biohazard hood.

**Figure 1:**
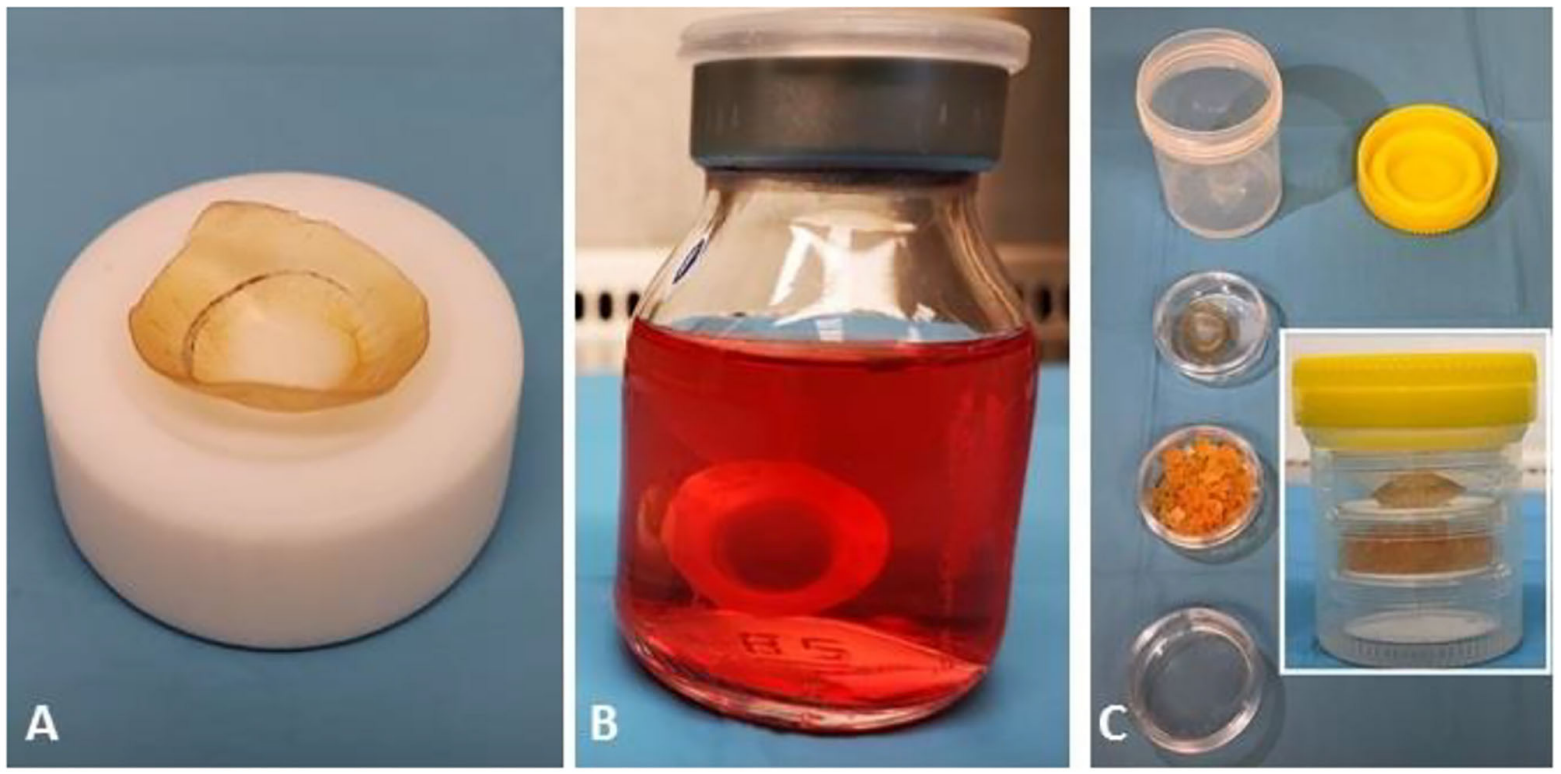
Appearance of the dehydrated and the rehydrated cornea and the preservation system. **(A)** Dehydration led to an opacification of the stroma and a yellowing of the cornea, with the scleral ring nearly indistinguishable from the stroma. **(B)** The rehydrated cornea has a distinctive appearance and thickness in the *Deswelling-Transport* solution. **(C)** Components of the system for maintaining the dehydrated cornea during storage and configuration of the final storage container.

The dehydrated corneas were transferred to a 25-mm sterile plastic Petri dish (Falcon 3001, Becton Dickinson, Waltham, MA, USA) and placed inside a 60-ml sterile single-use storage plastic jar (Nalgene, Nalge Nunc International, Rochester, NY, USA). The Petri dish containing the dehydrated cornea was positioned over two other 25-mm Petri dishes; the bottom one was empty and served simply to reduce the space in the jar, the middle one contained 2–6 mm silica gel beads (Chameleon, VWR Chemicals, Leuven, Belgium). The jar was closed with a screw top cap and stored at 4–6°C for up to 6 months (Figure 1), following the European rules for corneal tissues in the presence of silica gel (20).

On delivery to the surgical center, dehydrated corneas were transferred into sterile *Deswelling-Transport* solution (same composition as the *Cold* solution) to rehydrate and recover their physiological thickness for a minimum of 24 h before use.

### Sterility Testing

Sterility testing was achieved by sampling the *Storage* solution before the dehydration process, 6 days after the preservation in organ culture, and testing the *Deswelling-Transport* solution 24 h after the transfer of the dehydrated cornea prior to shipment to the surgical center. Microbial growth was screened in the media by means of two validated automated systems: Bactec 9240 (Becton-Dickinson, Franklin Lakes, NJ, USA) and HB&L (Alifax, Padua, Italy) (21).

Maintenance of sterile conditions during the dehydration process was assessed by a gelatin membrane filter (Gelatine Disposables, Sartorius, Gottingen, Germany) next to the Teflon base to collect airborne microbes that were then cultivated on blood agar plates for 7 days. In the event of positive results for microbial contamination of any of the tests, the cornea is discarded.

### FSL Incision

We used a Victus FSL (Technolas, Perfect Vision, Munich, Germany), which generates 290–550 fs pulses at 1,040 nm wavelength at 80 kHz repetition rate, to obtain ALK incisions with a straight-edge configuration. The rehydrated (RHD) corneas were mounted on an artificial anterior chamber (Network Medical Products, Coronet House, Ripon, UK) connected to a bottle of balanced salt solution (Alcon Laboratories Inc., Fort Worth, TX, USA) positioned at a height of 50 cm to maintain a pressure of 20 mmHg similar to that of the natural eye and sufficient to support the tissue.

Two lamellar incisions were made, a vertical rim incision and a horizontal bed incision, to assess the suturing of graft-to-receiver corneal edges and the quality of the graft-host interface, respectively. The FSL pulse energy was set at the level currently implemented to shape donor and receiver corneas for FSL-assisted ALK (Table 1) (22).

**Table 1:** FSL straight-edge configuration parameters for anterior lamellar keratoplasty.

**Common parameters**		
Anterior diameter	8.2 mm	Anterior diameter value
Depth ratio	79%	Ratio of cutting depth to pachimetry
Posterior diameter	7.5 mm	Posterior diameter value
**Vertical incision parameters—rim incision**		
Line spacing	2.0 μm	Distance between spots of adjacent lines
Spot spacing	4.0 μm	Distance between adjacent spots on a line
Side cut angle	70°	Angle between the corneal surface tangent and the rim cut
Top bonus	100 μm	Extension of the rim cut at the anterior side of the cornea to ensure exit of laser cut out of the eye
Bottom bonus	−10 μm	Extension of the rim cut inside the cornea to ensure that rim and bed cut overlap
Pulse energy	1.80 μJ	Energy level of each single pulse
**Horizontal incision parameters—bed incision**		
Line spacing	6.0 μm	Distance between spots of adjacent lines
Spot spacing	6.0 μm	Distance between adjacent spots on a line
Pulse energy	adjusted 0.8–1.8 μJ	Energy level of each single pulse

At the end of the procedure, the samples obtained under the cutting plane (posterior portion of the stroma, Descemet's membrane, and endothelium) served as bed samples, while those obtained from above the incision plane (epithelium, Bowman's layer, and anterior portion of the stroma) served as cap samples. All samples were examined using light microscopy, transmission electron microscopy (TEM) and scanning electron microscopy (SEM).

### Light Microscopy and TEM

The cornea samples were fixed by immersion in 2% glutaraldehyde in 0.1 M phosphate buffer, pH 7.4, for 2 h at 4°C. After three 5-min washes with phosphate buffer, they were post-fixed with 1% OsO_4_ diluted in 0.2 M potassium hexa-cyanoferrate for 1 h and then, after rinsing in 0.1 M phosphate buffer, dehydrated in graded concentrations of acetone, and embedded in a mixture of Epon and Araldite (Electron Microscopic Sciences, Fort Washington, PA, USA). Semi-thin sections of 1 μm thick were stained with toluidine blue and examined under an Olympus BX51 fluorescence microscope (Olympus, Tokyo, Japan) equipped with a digital camera (DKY-F58 CCD JVC, Yokohama, Japan). Digital images were analyzed with Image-Pro Plus 7.0 software (Media Cybernetics, Silver Spring, MD, USA).

For ultrastructural examination, ultrathin 70-nm sections were cut on an Ultracut E Ultramicrotome (Reichert-Jung, Heidelberg, Germany), contrasted with lead citrate, and observed on a Philips Morgagni 268 D transmission electron microscope (FEI Company, Eindhoven, The Netherlands), equipped with a MegaView 2 camera for the acquisition of digital images.

### Scanning Electron Microscopy

The corneal samples were fixed in 2% glutaraldehyde in 0.1 M phosphate for 2 h at 4°C, post-fixed with 1% OsO_4_ in the same buffer for 1 h at 4°C and dehydrated in graded concentrations of ethanol. They were then processed by critical point-drying (CPD 030, Balzers, Vaduz, Liechtenstein), mounted on stubs with colloidal silver, sputtered with gold by a MED 010 coater (Balzers), and examined with an XL30 scanning electron microscope (FEI Company).

## Results

**Figure 2:**
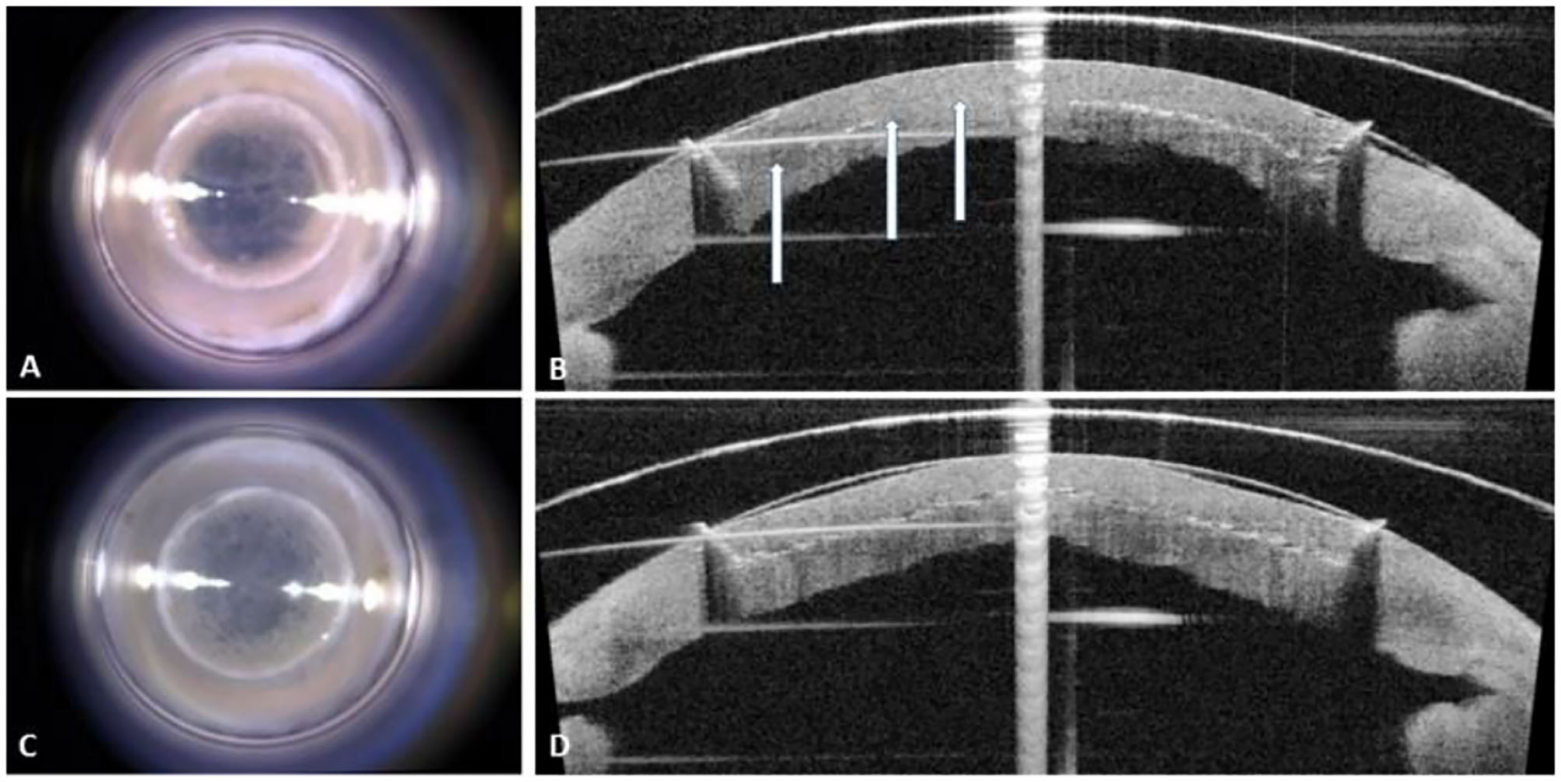
Surgical microscopy and integrated optical coherence tomography images of the rehydrated cornea following femtosecond laser (FSL) lamellar incision. **(A)** Frontal view of the failed incisions at 0.8 μJ. **(B)** Cut of the internal horizontal layer is largely absent (arrows). **(C)** Frontal view of the successful incisions. **(D)** Anterior, middle, and posterior views of the lamellar cut following straight-edge configuration.

Before FSL processing, the corneas displayed a normal physiological thickness. We shaped one rehydrated cornea by FSL at the pulse energy currently used to obtain a stroma lamellar cut for anterior keratoplasty, 1.80 and 0.80 μJ for rim and horizontal incision, respectively. The FSL procedure proved ineffective and was tricky to separate the lamellar cap from the posterior corneal bed because thin tissue bridges were formed in the corneal stroma at the horizontal plane cut (Figure 2A). To shape the subsequent RHD corneas, we increased the FSL energy for the horizontal incision to 1.0, 1.20, and 1.80 μJ, aligning rim and bed incision power. In all three cases, we obtained a clear-cut separation of the incised lamellar lenticule cap from the donor cornea bed, with the 1.2 μJ cut as good as the 1.8 μJ (Figure 2B), while residual stromal tissue bridges in the 1.0 μJ cut were recognized by the surgeon.

We successfully cut the CTRL corneas at 1.80 and 0.80 μJ for the rim and the bed incision, respectively (Figures 2C,D).

**Figure 3:**
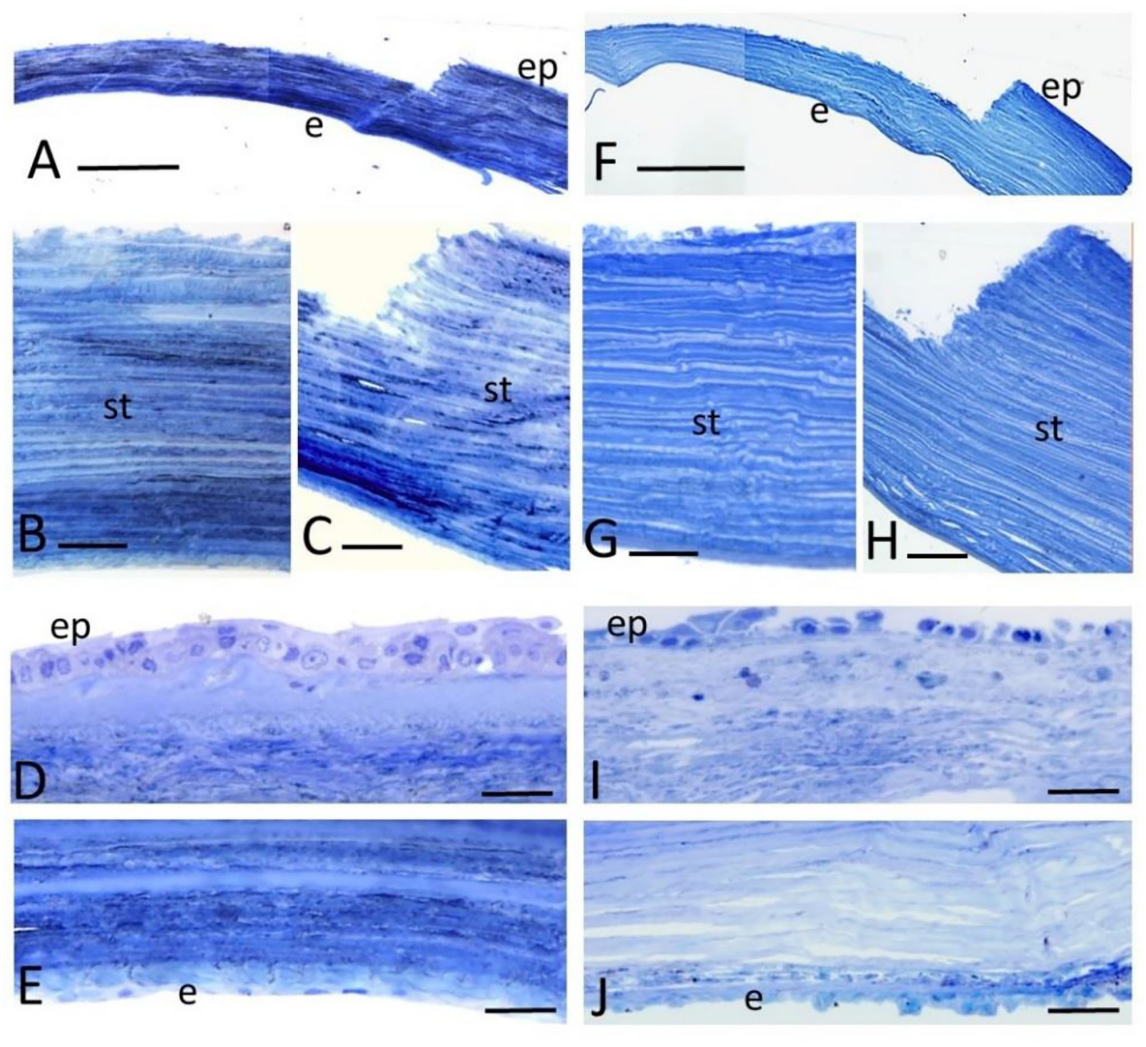
Serial light microscopy images of toluidine blue stained sections of the CTRL **(A–E)** and the RHD **(F–J)** cornea after lamellar incision. ep, denotes epithelium; st, stroma; and en, endothelium. Scale bar: **(A,F)** 500 μm; **(B,G)** 50 μm; **(C,H)** 100 μm; and **(D–J)** 50 μm.

Light microscopy of the toluidine blue-stained sections showed a well-preserved overall structure, well-defined incision borders, and distinct corneal layers (Figures 3A–C,F–H). The stromal collagen lamellae layers displayed a regular arrangement, with gaps in between lamellae in the corneal stroma in the CTRL and the RHD cornea. Compared with the CTRL cornea, the uppermost superficial epithelial layers of the RHD cornea were almost fully depleted (Figures 3D,I, respectively). The endothelium showed its typical organization, though detached from the Descemet's membrane in some areas in the CTRL and the RHD cornea (Figures 3E,J, respectively).

**Figure 4:**
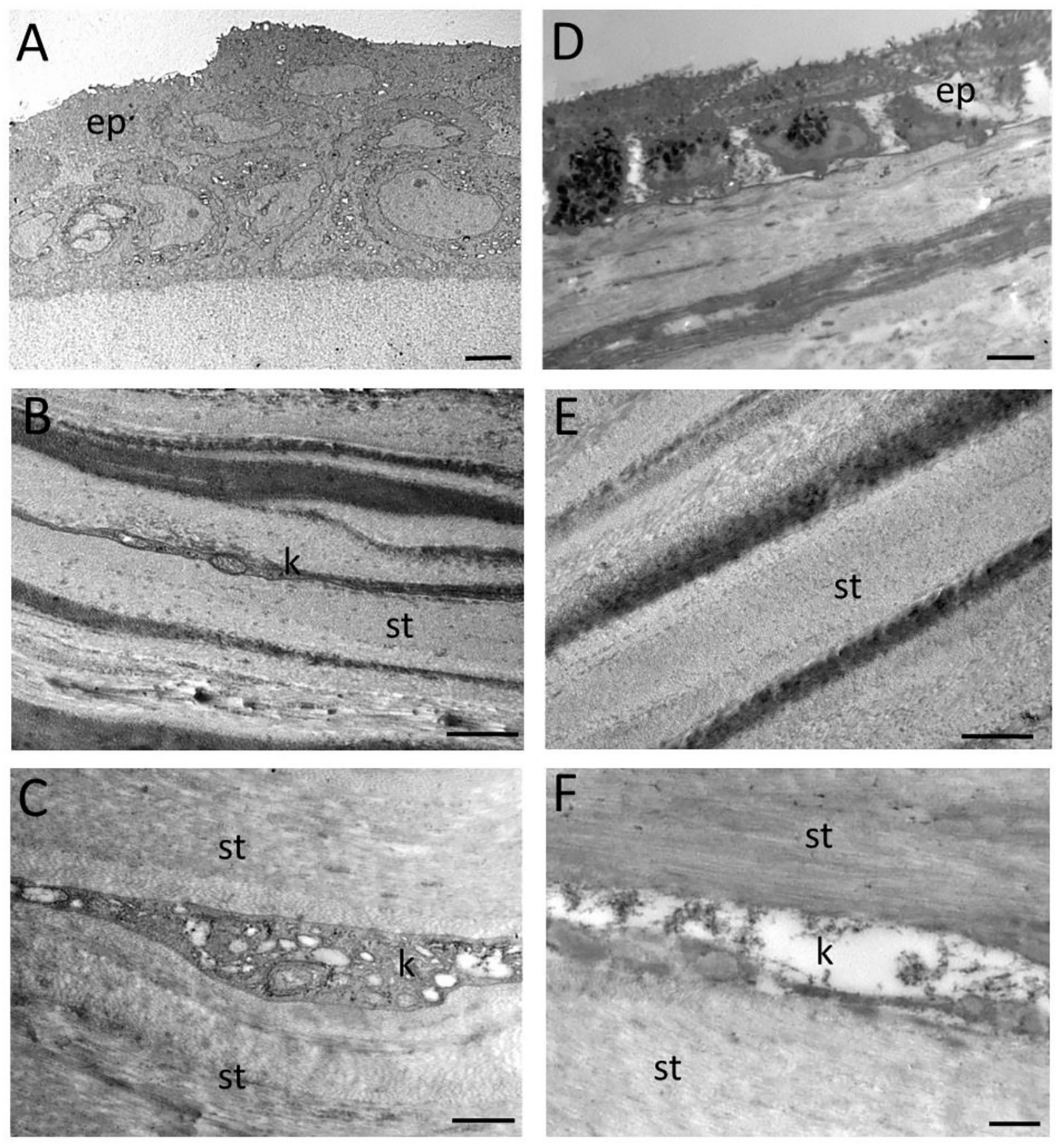
Transmission electron micrographs showing the epithelial surface not involved by laser incision and the deep corneal stroma of the CTRL **(A–C)** and the RHD **(D–F)** cornea. ep denotes epithelium; st, stroma; and k, keratocyte. Scale bar: **(A,D)** 2 μm; **(B,E)** 1 μm; and **(C,F)** 500 nm.

Ultrastructural TEM revealed no signs of thermal or mechanical damage to the corneal structure after FSL incision in either the CTRL or the RHD cornea. The epithelial basal membrane and Bowman's layer maintained their integrity in the CTRL (Figure 4A) and the RHD cornea; however, only the columnar basal layer of the epithelium and cells separated by large spaces due to junction alteration were retrieved in the RHD cornea (Figure 4D). No changes were noted in the organization of the lamellar layers in the stroma; an alternating light and dark staining pattern highlighted regular disposition of the collagen lamellae (Figures 4B,E). Gaps between the lamellar architecture were detectable throughout the stroma, particularly in the RHD cornea. Keratocytes were normal in structure and regularly distinguishable between the stroma lamellae in the CTRL cornea (Figure 4C), but sparse electron-dense with distinct organelles and damaged devoid keratocytes were detected in the RHD cornea (Figure 4F).

**Figure 5:**
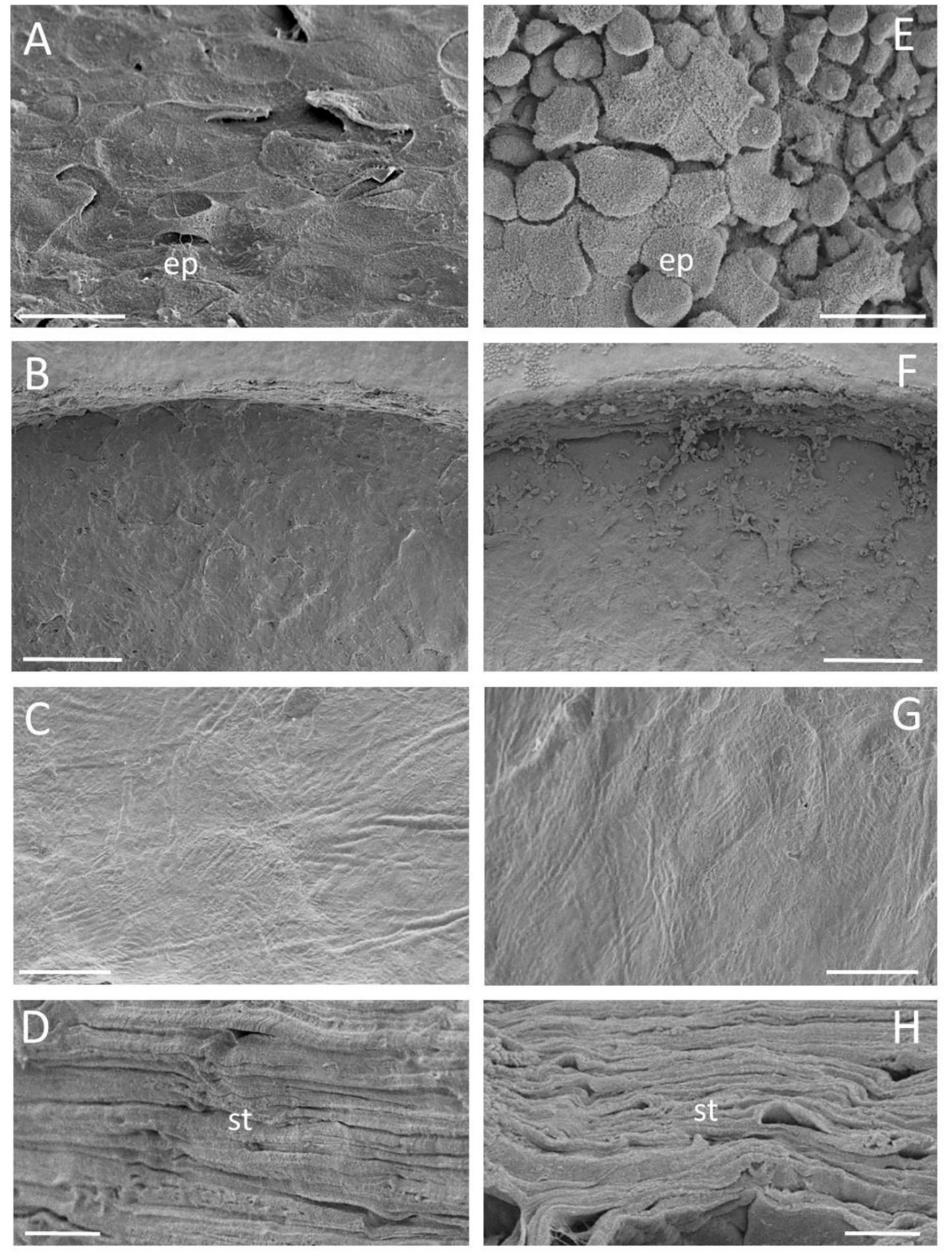
Scanning electron micrographs showing the epithelial surface not involved by laser incision **(A,E)** and the stromal surface after vertical and horizontal cuts in the CTRL **(B–D)** and the rehydrated **(F–H)** cornea. No differences were found between 1.2 and 1.8 μJ pulse energy horizontal incisions. Scale bar: **(A,E)** 20 μm; **(B,F)** 200 μm; **(C,G)** 50 μm; and **(D,H)** 10 μm.

Comparable results were obtained with SEM, which showed that the corneal surface not involved by the incision was covered with a proper multilayered intact epithelium in the CTRL cornea (Figure 5A), whereas that of the RHD cornea was devoid of the uppermost superficial epithelial layers, and large spaces were noted among the residual basal epithelial columnar cells (Figure 5E). A regular cutting surface with slightly irregular organization of the collagen lamellae was observed in the vertical incision in the CTRL (Figures 5B,C) and the RHD cornea (Figures 5F,G). The lamellar arrangement at the stroma appeared regular and smooth in both the CTRL and the RHD cornea (Figures 5D,H, respectively).

## Discussion

This study showed that air-dried corneas rehydrated in a solution containing dextran kept their overall corneal architecture, such as the Bowman's layer, endothelial basal membranes, and the organization of the stromal layer. Changes occurred in the spaces in between the collagen lamellae, without major alteration in corneal thickness, while some features at the cellular level appeared lost or altered.

Compared with the fresh cornea, the RHD cornea maintained epithelial basement membrane and the columnar basal epithelial cells only; the endothelial cells layer showed depletion in both fresh and RHD, a condition likely resulting from the manipulation during mounting of the cornea onto the artificial anterior chamber for FSL ablation; and the anatomical structure and vitality of the stromal keratocytes were found to have deteriorated.

The maintenance of integrity of the epithelial basement membrane and Bowman's layer is essential for effective epithelial renewal after grafting, when the donor epithelium is replaced by that of the receiver.

Degeneration of keratocytes with necrotic and apoptotic changes due to the dehydration process is expected. It has been observed with ultrastructural evaluation in dehydrated corneas after lyophilization or cryopreservation, with or without the use of a cryoprotective agent (10, 23). For this reason, it was not necessary to include a control group to compare air-drying with other procedures for dehydrating corneal tissue. However, the absence of vital keratocytes in RHD corneas intended for ALK or reconstructive keratoplasty is not an issue, as migration of host keratocytes into the donor stroma with the promotion of gradual replacement of keratocytes and maintenance of the synthesis of new collagen is expected (24).

Finally, the vitality of endothelial cells is not an issue as only the anterior part of the cornea is intended for transplantation.

We observed no negative effects following rehydration with the *Deswelling-Transport* solution containing dextran, artifacts after dehydration, metallization for TEM and SEM observations, or negative FSL sequelae. What we did find significant for application in FSL-assisted keratoplasty is that the laser pulse energy needs to be increased compared with fresh corneas used as control to obtain the horizontal stromal incision. Irregularities in the fibril arrangement in the RHD cornea, albeit slight compared with the CTRL cornea, and focal regions of irregularly spaced collagen lamellae induced by dehydration-rehydration may increase Rayleigh scattering resulting in reduced bean quality and depth of penetration (25).

Since the light scattering mechanism is wavelength-dependent, increasing the wavelength could improve incision quality, as our findings show. Clinical systems, such as the Victus 80 KHz FSL use a wavelength of 1,040 nm, which assures satisfactory incision penetration and prevents unwanted thermal side effects. The pulse energy can be adjusted, however, and we improved the horizontal cut by raising it from 0.8 to 1.0 μJ and 1.2 μJ. At the high pulse energies of 1.2 and 1.8 μJ, we observed a remarkably smooth surface without major abnormalities, such as melted-like photo-disrupted collagen fibrils (26); the ultrastructural result ensures optical quality of the graft performed using corneas after dehydration-rehydration, and FSL parameters. A smooth surface generated by an effective FSL-assisted incision provides for an excellent interface and apposition of donor and recipient surfaces, with a clean incision border for suturing a graft-to-host edge in precise apposition. Such broad and close contact between donor and host parenchyma could further benefit keratocyte repopulation of the dehydrated tissue.

Our data show that dehydration by air-drying is associated with minor changes in corneal structure and that following rehydration corneas are a reliable source of tissues for FSL-assisted optical or reconstructive ALK.

Compared with other techniques for dehydrating corneal tissue, air-drying closely resembles the method currently applied for processing amniotic membrane (27), it is simple, cheap, and can be easily incorporated into routine eye bank protocols.

## Data Availability Statement

The raw data supporting the conclusions of this article will be made available by the authors, without undue reservation.

## Author Contributions

All authors listed have made a substantial, direct, and intellectual contribution to the work and approved it for publication.

## Conflict of Interest

The authors declare that the research was conducted in the absence of any commercial or financial relationships that could be construed as a potential conflict of interest.

## Publisher's Note

All claims expressed in this article are solely those of the authors and do not necessarily represent those of their affiliated organizations, or those of the publisher, the editors and the reviewers. Any product that may be evaluated in this article, or claim that may be made by its manufacturer, is not guaranteed or endorsed by the publisher.
